# A sex-stratified long-term clinical outcome analysis in coronary chronic total occlusion patients

**DOI:** 10.1186/s13293-020-00354-z

**Published:** 2021-01-08

**Authors:** Xuhe Gong, Li Zhou, Xiaosong Ding, Hongwei Li, Hui Chen

**Affiliations:** 1 Department of Cardiology, Cardiovascular Center, Beijing Friendship Hospital, Capital Medical University, Road 95 Yongan Xicheng District, Beijing, 100050 People’s Republic of China; 2 Department of Internal Medicine, Medical Health Center, Beijing Friendship Hospital, Capital Medical University, Beijing, 100050 People’s Republic of China; 3 Beijing Key Laboratory of Metabolic Disorder Related Cardiovascular Disease, Beijing, 100069 People’s Republic of China

**Keywords:** Chronic total occlusions, Percutaneous coronary intervention, Sex-based differences, Revascularization

## Abstract

**Background:**

Differences in outcomes for women and men after percutaneous coronary intervention (PCI) in chronic total occlusion (CTO) patients remain controversial. Herein, we compared the clinical outcomes by sex in CTO patients undergoing PCI.

**Methods:**

A total of 563 consecutive patients (19% women) who were diagnosed with CTO at a single center in China from June 2017 to December 2019 were included in this study. Three hundred patients were revascularized by PCI, and 263 were not revascularized. The clinical outcomes of these patients stratified by sex were examined. The primary endpoints included the risk of major adverse cardiovascular and cerebrovascular events (MACCE); the secondary endpoint was cardiac death; hazard ratios were generated using multivariable Cox regression.

**Results:**

Women represented 19% of the cohort (107/563 patients). Women have lower mean body mass index (BMI) and abdominal circumference compared with men; however, the proportion of hypertension, diabetes, and previous coronary heart disease is higher in female patients. At 2-year follow-up, there were no differences between men and women for MACCE (15.8% vs 20.6%, *p* = 0.234) and cardiac death (3.1% vs 5.6%, *p* = 0.202). Predictors of CTO recanalization revealed that age < 65 years, absence of prior CABG, no history of DM, and non-triple vessel were predictors of CTO recanalization. Sex did not predict recanalization in this regression model. Successful CTO PCI was associated with reduced MACCE.

**Conclusion:**

Our study suggests an equal benefit of CTO recanalization with a marked reduction in MACCE in women and men alike. Further dedicated studies are needed to confirm these findings.

## Introduction

Chronic total occlusions (CTOs) are an important subgroup of coronary lesions; with new techniques and devices being developed, the success rate of PCI opening CTO lesions is gradually increasing. Successful percutaneous coronary intervention (PCI) of CTO has been shown to improve left ventricular (LV) systolic function, reduce angina, increase exercise capacity, and a potential benefit on mortality [1]. However, due to the low number of women enrolled in clinical trials, the literature regarding sex-related differences in CTO outcomes becomes scarce. Indeed, men are more likely to suffer from ischemic heart disease, while women mainly develop ischemic heart disease in the postmenopausal period due to the protective effects of estrogens, therefore, much later than in men [2]. Moreover, the prevalence of CTO is similar in women and men [3]. However, the relationship between sex and CTO PCI outcomes remains controversial. Thus, we present here the results of our observational study comparing the impact of CTO percutaneous revascularization in men versus women.

## Methods

### Study population

We retrospectively evaluated consecutive 592 patients with CTO from Beijing Friendship Hospital Database (CBD) who were admitted to the department of cardiology from June 2017 to October 2019. The local institutional review board at our hospital approved the study protocol, and this study was in accord with the Declaration of Helsinki.

Patients were included in the study if they fulfilled the following criteria: CTO was defined as a 100% occlusion with antegrade intraluminal TIMI (thrombolysis in myocardial infarction) flow grade of 0 and clinical or angiographic evidence of occlusion duration > 3 months [4]. In total, 29 patients were excluded from this study because they had complications of active infectious disease, chronic inflammatory disease, rheumatologic disease, malignant tumor, or treated by CABG (within 3 months after the CTO diagnosis). Finally, a total of 563 patients were included in the study.

### Data collection, endpoints, and definitions

The patients’ demographic information and cardiovascular risk factors including previous coronary heart disease, hypertension, diabetic mellitus, dyslipidemia, chronic kidney disease, peripheral vascular disease, and smoking history were retrospectively collected from medical records. Body mass index (BMI) was calculated by dividing weight in kilograms by height in meters squared (kg/m^2^). The estimated glomerular filtration rate (eGFR) was calculated by standard calculations (GFR based on levels of creatinine [GFR(epi)]) [5]. The left ventricular ejection fraction (LVEF) was determined using 2-dimensional echocardiography during the index hospitalization.

The primary endpoints were major adverse cardiovascular and cerebrovascular events (MACCE), which were defined as the composite of death, recurrent myocardial infarction, target lesion revascularization, re-hospitalization, heart failure, and stroke during follow-up. The secondary endpoint was cardiac death. Death that could not be attributed to a non-cardiac etiology was considered cardiac death. MI was defined according to the latest definition provided by the European Society of Cardiology [6]. CTO revascularization (CTO-R) was defined as final residual stenosis less than 20%, with TIMI grade ≥ 2 flow on visual assessment.

Follow-up information discharged from the hospital was obtained by planned telephone interviews. All patients were evaluated by clinical visit or by phone at 1, 6, and 12 months, and annually thereafter.

### Statistics

Continuous variables are expressed as mean ± standard deviation or median with interquartile range, and one-way analysis of variance was used to compare differences between continuous variables. Categorical variables are expressed as percentages and were analyzed using the Pearson’s χ^2^ test or Fisher’s exact test of variance. The cumulative incidence was estimated by the Kaplan-Meier method, and differences between groups were assessed by the log-rank test. Cox regression was used to estimate relative risks among groups of patients adjusted according to sex and procedural characteristic. All statistical tests were two-tailed, with statistical significance defined as a *p* value of < 0.05. All analyses were performed by using SPSS 25 (SPSS lnc, Chicago, IL, USA); Kaplan–Meier survival curves were generated with the use of the GraphPad Prism software (version 5; GraphPad, lnc, San Diego, CA).

## Results

### Baseline characteristics between women and men with CTOs

Patients stratified by sex and baseline characteristics are summarized in Table 1. Women constituted 19% of the cohort (107/563 patients). Women were significantly older and had more frequently diabetes mellitus (64.5% vs 41.7%, *p* < 0.001), hypertension (86% vs 70.4%, *p* = 0.001), and coronary artery disease (72% vs 58.6%, *p* = 0.01). Women and men had a similar prevalence of chronic kidney disease, peripheral vascular disease, and stroke, and history of PCI and CABG. Men were more likely to be smokers and drinker. Abdominal circumference and BMI were larger in men. However, compared with men, women had higher CHOL, LDL-C, and NT-proBNP, and lower estimated glomerular filtration rate (eGFR) (all *p* < 0.05).

**Table 1:** Comparison of the baseline clinical characteristics among patients

	Total (***n*** = 563)	Men (***n*** = 456)	Women (***n*** = 107)	***p***
**Age (mean ± SD), years**	**64.7 ± 10.5**	**63.6 ± 10.6**	**69.5 ± 8.7**	**< 0.001**
Hospitalization days	7 (5, 9)	7 (5, 8)	7 (5, 9)	0.425
**BMI (mean ± SD), kg/m**^**2**^	**26.03 ± 3.42**	**26.19 ± 3.42**	**25.35 ± 3.35**	**0.022**
**AC (mean ± SD), cm**	**93.51 ± 9.86**	**94.3 ± 9.64**	**90.07 ± 10.11**	**< 0.001**
**Smoke**	**371 (65.9)**	**349 (76.5)**	**22 (20.6)**	**< 0.001**
**Drink**	**113 (20.1)**	**112 (24.6)**	**1 (0.9)**	**< 0.001**
**Hypertension**	**413 (73.4)**	**321 (70.4)**	**92 (86)**	**0.001**
**Diabetes mellitus**	**259 (46)**	**190 (41.7)**	**69 (64.5)**	**< 0.001**
Hyperlipidemia	319 (56.7)	255 (55.9)	64 (59.8)	0.465
**CAD**	**344 (61.1)**	**267 (58.6)**	**77 (72)**	**0.01**
Previous MI	126 (22.4)	98 (21.5)	28 (26.2)	0.296
Heart failure	8 (1.4)	5 (1.1)	3 (2.8)	0.179
Chronic kidney disease	34 (6)	26 (5.7)	8 (7.5)	0.488
Peripheral vascular disease	42 (7.5)	32 (7)	10 (9.3)	0.409
Stroke	121 (21.5)	92 (20.2)	29 (27.1)	0.116
History of PCI	176 (31.3)	141 (30.9)	35 (32.7)	0.719
History of CABG	23 (4.1)	21 (4.6)	2 (1.9)	0.198
**CHOL(mmol/L)**	**4.02 ± 106**	**3.96 ± 1.04**	**4.32 ± 1.11**	**0.002**
**LDL-C(mmol/L)**	**2.28 ± 0.75**	**2.24 ± 0.73**	**2.42 ± 0.79**	**0.028**
**eGFR(ml/min)**	**83.4 ± 22.11**	**84.68 ± 21.56**	**77.95 ± 23.67**	**0.005**
**NT-proBNPmax (pg/ml)**	**370 (106.5, 1337.5)**	**328 (94.53, 1196.75)**	**582 (135.5, 2152.5)**	**0.044**

Values are *n* (%), mean ± SD, or median with interquartile range

*AC* abdominal circumference, *CAD* coronary artery disease, *PCI* percutaneous coronary intervention, *CABG* coronary artery bypass graft, *CHOL* cholesterol, *LDL-c* low-density lipoprotein cholesterol, *NT-proBNP* N-terminal pro-brain natriuretic peptide, *eGFR* estimated glomerular filtration rate

### Echocardiography results and angiographic characteristics

**Fig. 1:**
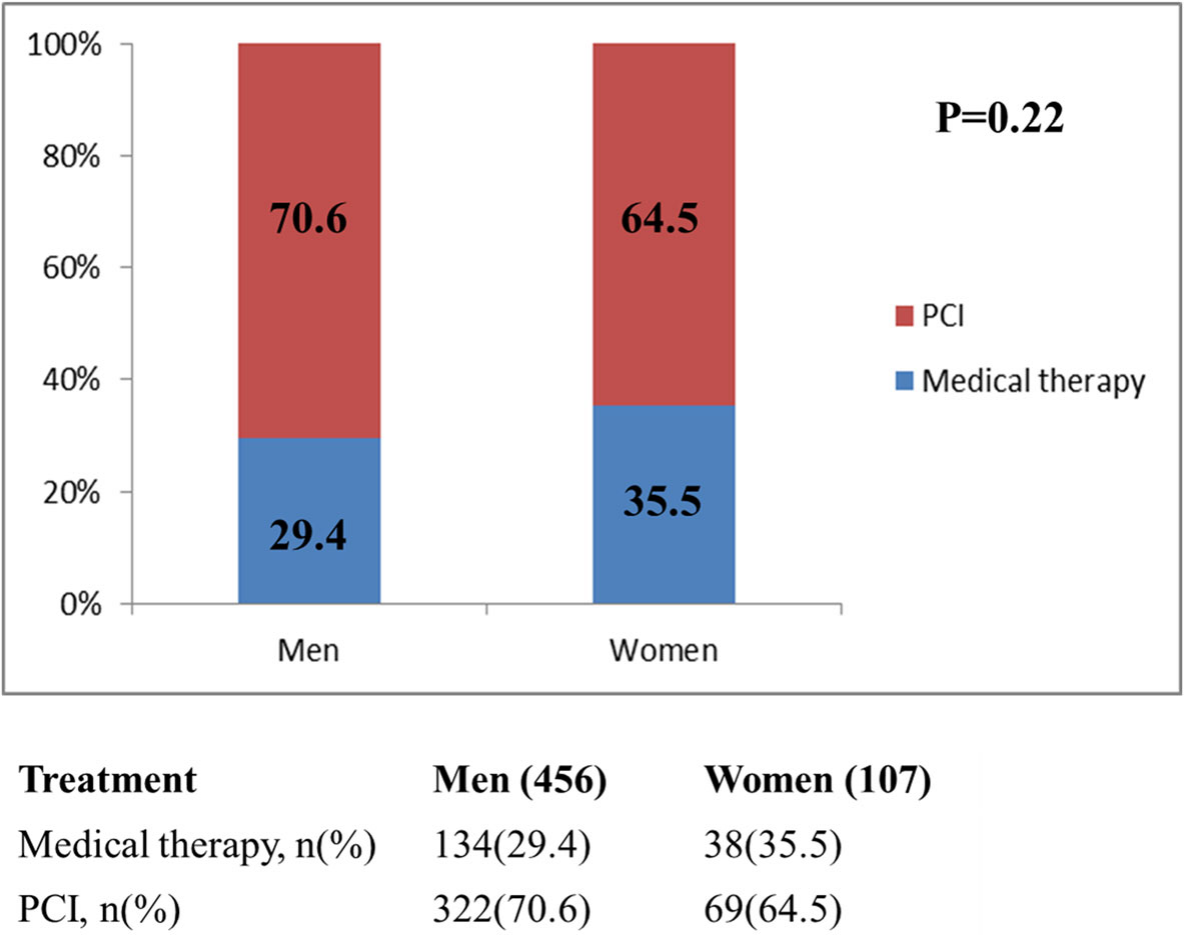
CTO treatment decisions according to gender

The echocardiography results and angiographic characteristics between sexes are shown in Table 2. The left ventricular end-diastolic dimension (LVEDD) was higher in men (5.31 ± 0.63 vs 5 ± 0.47, *p* < 0.001). There was no difference between left ventricular ejection fraction (LVEF) and FS (fraction shortening) for men and women. In coronary angiography, there were no significant differences in the CTO-related artery distribution between genders; women and men had a similar prevalence of three-vessel disease, left main coronary artery disease, and in-stent restenosis (ISR). The initial treatment strategies between different genders are visually shown in Fig. 1; the rates of PCI were similar between gender (70.6 vs 64.5, *p* = 0.22). Moreover, CTO recanalization rate was similar between women and men (54.4 vs 48.6%, *p* = 0.28). Sex was not associated with CTO recanalization (odds ratio 0.999, confidence interval 0.633–1.574, *p* = 0.995) in multivariate analysis (Table 3). The results revealed that age < 65 years, absence of prior CABG, no history of DM, and non-triple vessel were predictors of CTO recanalization.

**Table 2:** Angiographic characteristics and echocardiography results stratified by gender

	Total (***n*** = 563)	Men (***n*** = 456)	Women (***n*** = 107)	***p***
**LVEDD (mean ± SD), cm**	**5.25 ± 0.62**	**5.31 ± 0.63**	**5 ± 0.47**	**< 0.001**
LVEF (mean ± SD)	0.6 ± 0.11	0.59 ± 0.1	0.61 ± 0.11	0.359
FS (mean ± SD)	0.32 ± 0.07	0.32 ± 0.07	0.33 ± 0.07	0.242
Target CTO vessel, *n* (%)				0.816
LAD	157 (27.9)	131 (28.7)	26 (24.3)	
LCX	143 (25.4)	114 (25)	29 (27.1)	
RCA	206 (36.6)	166 (36.4)	40 (37.4)	
≥2 vessel	57 (10.1)	45 (9.9)	12 (11.2)	
ISR	37 (6.6)	26 (5.7)	11 (10.3)	0.085
Three-vessel disease	523 (92.9)	423 (92.8)	100 (93.5)	0.801
LM disease	107 (19)	86 (18.9)	21 (19.6)	0.856
CTO revascularization	300 (53.3)	248 (54.4)	52 (48.6)	0.28

Values are *n* (%), mean ± SD, or median with interquartile range

*LVEDD* left ventricular end-diastolic dimension, *LVEF* left ventricular ejection fraction, *FS* fraction shortening, *LAD* left anterior descending coronary artery, *LCX* left circumflex coronary artery, *RCA* right coronary artery disease, *ISR* in-stent restenosis, *LM disease* left main coronary artery disease

**Table 3:** Predictors of CTO recanalization

Variable	Odds ratios	95% CI	***p***
Sex (female vs male)	0.999	0.633–1.574	0.995
Age ≥ 65 years	0.532	0.372–0.761	0.001
History of DM	0.69	0.482–0.987	0.042
History of CKD	0.397	0.23–1.079	0.077
Prior CABG	0.171	0.049–0.597	0.006
LM disease	0.659	0.416–1.044	0.076
Three-vessel disease	0.397	0.185–0.852	0.018

*DM* diabetes mellitus, *CKD* chronic kidney disease, *CABG* coronary artery bypass graft

### Clinical outcomes

The clinical outcomes in men and women are shown in Table 4. There were no differences between men and women in all-cause mortality (3.7% vs 6.5%, *p* = 0.195), cardiac death (3.1% vs 5.6%, *p* = 0.202), re-hospitalization (13.6% vs 16.8%, *p* = 0.39), heart failure (2.2% vs 1.9%, *p* = 0.835), target vessel revascularization (1.1% vs 1.9%, *p* = 0.516), or MACCE (15.8% vs 20.6%, *p* = 0.234). However, women had significantly higher rates of stroke than men (2.8% vs 0.2%, *p* < 0.004).

**Table 4:** Comparison of clinical outcome between different sexes

Event, ***n*** (%)	Men (***n*** = 456)	Women (***n*** = 107)	***p***
MACCE	72 (15.8)	22 (20.6)	0.234
All-cause mortality	17 (3.7)	7 (6.5)	0.195
Cardiac death	14 (3.1)	6 (5.6)	0.202
Re-hospitalization	62 (13.6)	18 (16.8)	0.39
Heart failure	10 (2.2)	2 (1.9)	0.835
Target vessel revascularization	5 (1.1)	2 (1.9)	0.516
Recurrent myocardial infarction	8 (1.8)	3 (2.8)	0.48
Stroke	1 (0.2)	3 (2.8)	0.004

*MACCE* major adverse cardiac and cerebrovascular events

**Fig. 2:**
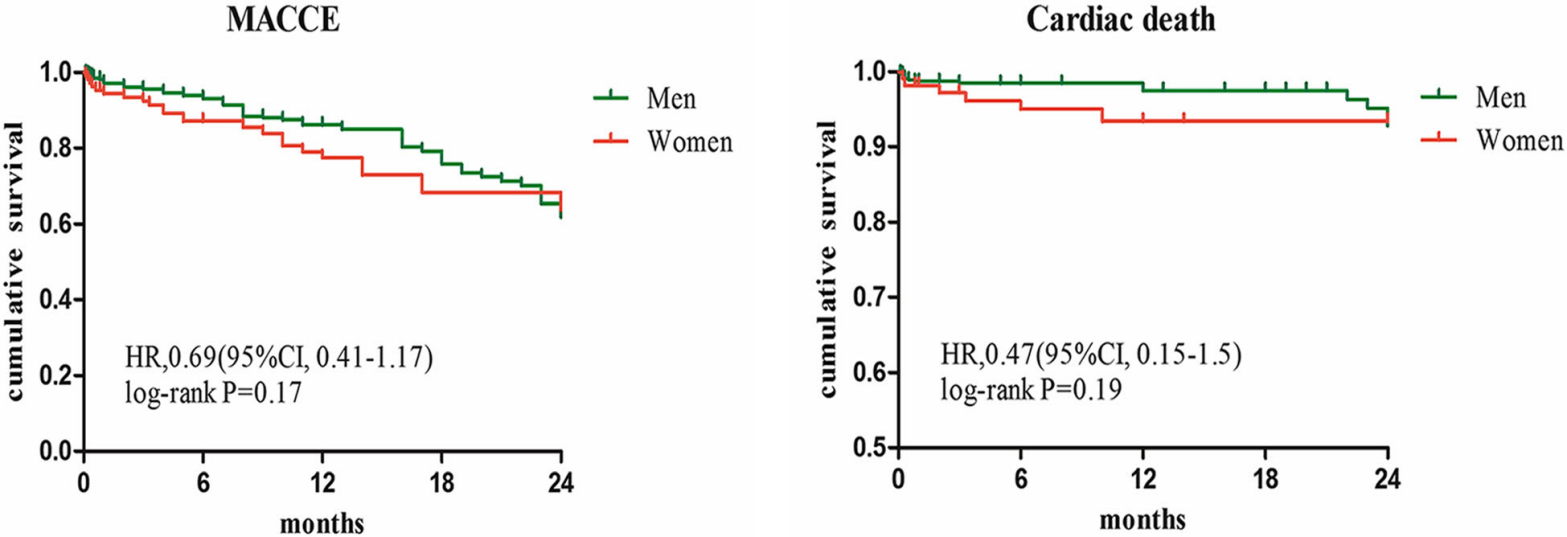
Kaplan-Maier curves for MACCE and cardiac death between female and male group

**Fig. 3:**
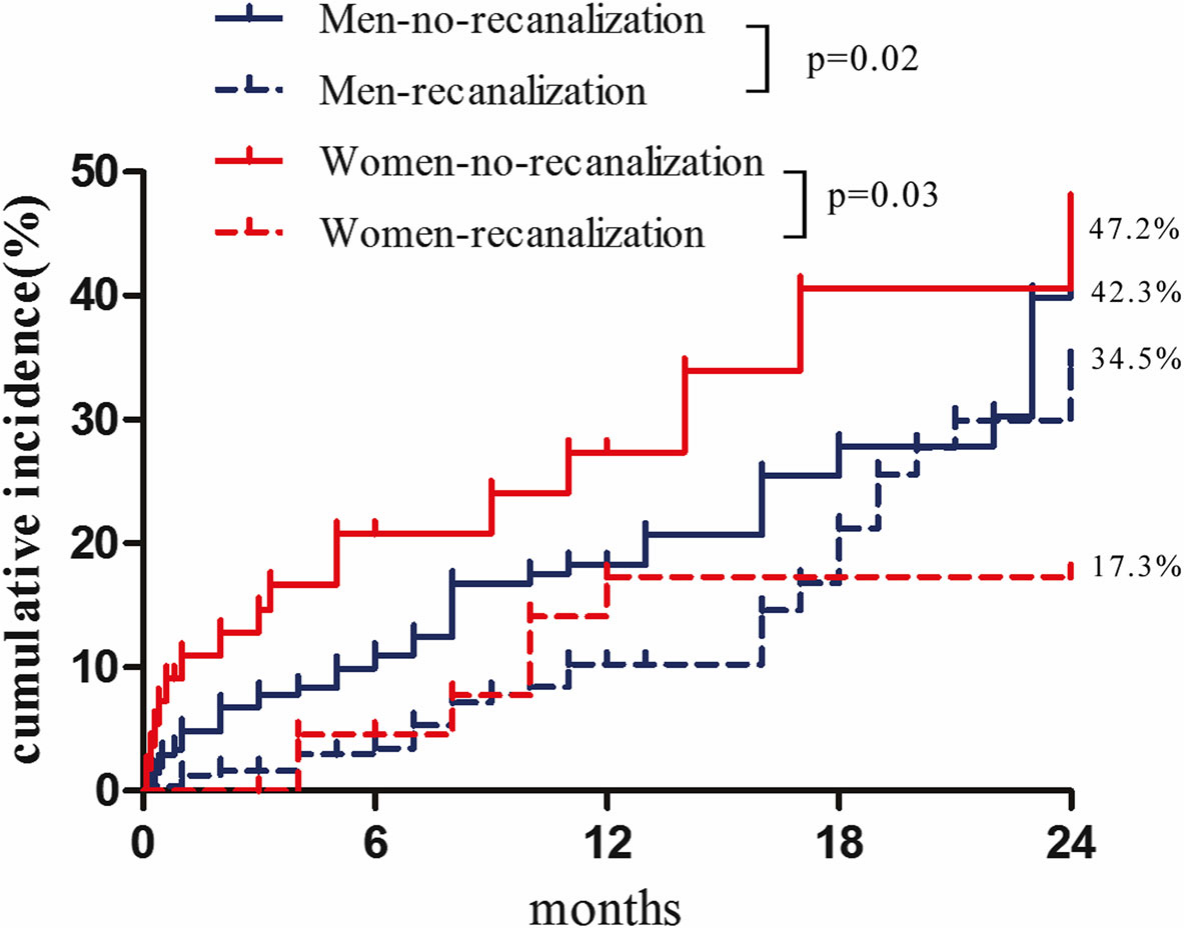
MACCE stratified by sex and CTO recanalization. Cumulative MACCE rates of CTO recanalization or no-recanalization in male and female patients

Kaplan-Maier curves according to the MACCE-free survival and cardiac death-free survival also revealed no difference between men and women group (Fig. 2). Moreover, Kaplan-Meier survival curves for cumulative MACCE in male and female patients are displayed in Fig. 3, showing again a survival benefit in CTO revascularized patients of both sexes.

**Fig. 4:**
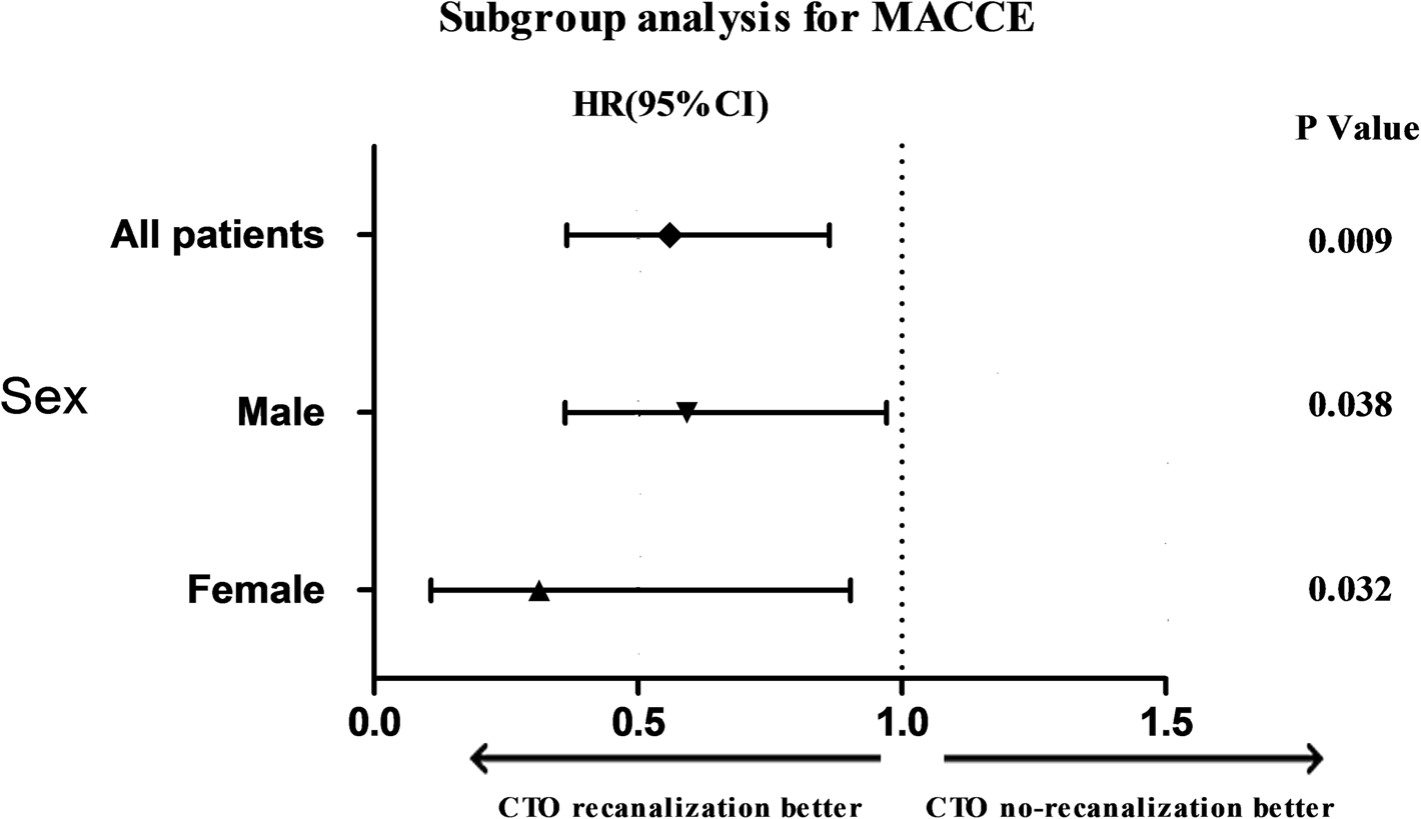
Sex subgroup analysis for MACCE

Multivariate predictors of cardiac death and MACCE are reported in Table 5. For MACCE, the final multivariable mode included LVEF < 0.5, eGFR < 60 ml/min, and CTO recanalization. For cardiac death, the final multivariable mode included LVEF < 0.5, eGFR < 60 ml/min, LM disease, and CTO recanalization. Overall, CTO recanalization was a protected predictor of cardiac death (HR 0.28, 95% CI 0.091–0.88, *p* = 0.03) and MACCE (HR 0.56, 95% CI 0.365–0.863, *p* = 0.009). The survival benefit after CTO recanalization was similar in men and women (Fig. 4).

**Table 5:** Multivariate Cox hazard model predicting MACCE and cardiac death

Variable	MACCE		Cardiac death	
	HR (95%CI)	*p*	HR (95%CI)	*p*
Age ≥ 65 years	0.83 (0.53–1.301)	0.42	0.41 (0.143–1.164)	0.09
Sex^a^	1.4 (0.838–2.334)	0.2	2.1 (0.709–6.216)	0.18
HT	0.92 (0.571–1.464)	0.71	1.21 (0.403–3.628)	0.73
DM	0.81 (0.526–1.233)	0.32	0.71 (0.271–1.857)	0.49
HP	0.91 (0.604–1.377)	0.66	0.97 (0.376–2.479)	0.94
EF < 0.5	2.11 (1.326–3.365)	0.002	5.12 (2.053–12.742)	< 0.001
eGFR < 60 ml/min	2.06 (1.223–3.484)	0.007	5.33 (1.998–14.237)	0.001
LM disease	1.16 (0.703–1.913)	0.56	3.69 (1.466–9.296)	0.006
CTO recanalization	0.56 (0.365–0.863)	0.009	0.28 (0.091–0.88)	0.03

^a^For women vs. men; multivariable Cox hazards regression analysis performed in the overall CTO patients

## Discussion

The present study provides new data on the sex-related differences in CTO patients in a large Chinese cardiovascular center. We found that there were no sex differences in CTO recanalization, cardiac death, or MACCE. CTO-PCI was associated with a significantly lower rate of cardiac death and MACCE in men and women alike.

In the present cohort, 19% of all CTO patients were performed in women. This was consistent with the results of previous studies that women were under-represented in CTO patients [7]. Moreover, women were significantly older and had more frequently diabetes mellitus, hypertension, and the history of coronary artery disease. Women also had lower eGFR levels, as previously reported [8]. In contrast, blood lipid levels are higher in female patients in our study.

Previous study has reported that revascularization therapy to the CTO artery was performed significantly less in women [3, 9]. Unlike previous research, our study found that initial treatment strategies choosing PCI or medical therapy between different genders were similar. Moreover, CTO recanalization rate was also comparable in male and female patients; gender does not affect CTO recanalization; the predictive factors of CTO recanalization included age < 65 years, absence of prior CABG, no history of DM, and non-triple vessel in this study. The treatment decision for a patient with CTO is closely related to the severity of concomitant coronary artery disease; in our study population, there was no difference in the severity of coronary artery disease and the proportion of left main disease, and three-vessel disease was similar between male and female patients. We speculated that this could account for the no difference in treatment strategies between men and women.

According to a recently published global expert consensus, ischemic symptom improvement is the primary indication for CTO-PCI [10]. Whether CTO-PCI can reduce the risk for MACE remains controversial. In our experience, both men and women benefited from improved survival after successful recanalization; which was consistent with previously published data by Akodad et al. [11]. Our results showed that successful CTO PCI is associated with a statistically significant improvement in cardiac death and MACCE; CTO recanalization was a protected predictor in all population. More importantly, the incidence of MACCE events in the female CTO revascularized patients also decreased, with the rapid development of the CTO field; better management of CTO in all patients is paramount. Female CTO patient included in previous studies accounted for a small proportion; more attention should be paid to female CTO patients in the future.

## Limitations

A major limitation of this study is that it is an observational and single center study; moreover, there may be a selection bias toward who may be referred for PCI. Third, because China population was exclusively included in our study, it is uncertain whether these findings can be applied to other ethnic groups or research institute with different patient characteristics and procedural strategies.

## Conclusion

In summary, women derived the same benefit from CTO recanalization as men in this single center observational study. The CTO recanalization and MACCE in females were the same as in men. These findings have important clinical implications. We should strengthen our attention to female CTO patients.

### Perspectives and significance

It is well documented that gender differences existed in the presentation and outcomes of patients with coronary artery disease. However, the literature regarding sex-related differences in CTO outcomes becomes scarce due to the low number of women enrolled in clinical trials. We sought to examine the clinical outcomes by sex in Chinese CTO patients. Women were significantly older and had more frequently diabetes mellitus, hypertension, and the history of coronary artery disease. There were no sex differences in CTO recanalization, cardiac death, or MACCE. Women derived the same benefit from CTO recanalization as men. Large-scale randomized clinical trials focusing on percutaneous revascularization of CTO are needed to reveal the sex-related differences in the future.

## References

[1] Tajti P, Burke MN, Karmpaliotis D (2018). Update in the percutaneous management of coronary chronic total occlusions. JACC Cardiovasc Interv.

[2] Heras M (2006). Ischemic heart disease in women: clinical presentation, non-invasive testing and management of acute coronary syndromes. Revista espanola de cardiologia.

[3] Wolff R, Fefer P, Knudtson ML (2016). Gender differences in the prevalence and treatment of coronary chronic total occlusions. Catheterization Cardiovasc Interv.

[4] Di Mario C, Werner GS, Sianos G (2007). European perspective in the recanalisation of chronic total occlusions (CTO): consensus document from the EuroCTO Club. EuroIntervention.

[5] Lambrinoudaki I, Tourlakis D, Armeni E (2015). Variations in glomerular filtration rate are associated with subclinical atherosclerosis in healthy postmenopausal women. Menopause (New York).

[6] Thygesen K, Alpert JS, Jaffe AS (2018). Fourth universal definition of myocardial infarction (2018). Circulation.

[7] Claessen BE, Chieffo A, Dangas GD (2012). Gender differences in long-term clinical outcomes after percutaneous coronary intervention of chronic total occlusions. J Invasive Cardiol.

[8] De Boer SP, Roos-Hesselink JW, Van Leeuwen MA (2014). Excess mortality in women compared to men after PCI in STEMI: an analysis of 11,931 patients during 2000-2009. Int J Cardiol.

[9] Sharma V, Wilson W, Smith W (2017). Comparison of characteristics and complications in men versus women undergoing chronic total occlusion percutaneous intervention. Am J Cardiol.

[10] Brilakis ES, Mashayekhi K, Tsuchikane E (2019). Guiding principles for chronic total occlusion percutaneous coronary intervention. Circulation.

[11] Akodad M, Spaziano M, Garcia-Alonso CJ (2019). Is sex associated with adverse outcomes after percutaneous coronary intervention for CTO?. Int J Cardiol.

